# Analysis of the potential biological mechanisms of diosmin against renal fibrosis based on network pharmacology and molecular docking approach

**DOI:** 10.1186/s12906-023-03976-z

**Published:** 2023-05-13

**Authors:** Wen-Man Zhao, Zhi-Juan Wang, Rui Shi, Yuyu Zhu, Xun-Liang Li, De-Guang Wang

**Affiliations:** 1 Department of Nephrology, the Second Affiliated Hospital of Anhui Medical University, Hefei, China; 2 Institute of Kidney Disease, Inflammation & Immunity Mediated Diseases, the Second Affiliated Hospital of Anhui Medical University, Hefei, China

**Keywords:** Diosmin, Molecular docking, Pharmacological mechanisms, Renal fibrosis

## Abstract

**Background:**

Interstitial fibrosis is involved in the progression of various chronic kidney diseases and renal failure. Diosmin is a naturally occurring flavonoid glycoside that has antioxidant, anti-inflammatory, and antifibrotic activities. However, whether diosmin protects kidneys by inhibiting renal fibrosis is unknown.

**Methods:**

The molecular formula of diosmin was obtained, targets related to diosmin and renal fibrosis were screened, and interactions among overlapping genes were analyzed. Overlapping genes were used for gene function and KEGG pathway enrichment analysis. TGF-β1 was used to induce fibrosis in HK-2 cells, and diosmin treatment was administered. The expression levels of relevant mRNA were then detected.

**Results:**

Network analysis identified 295 potential target genes for diosmin, 6828 for renal fibrosis, and 150 hub genes. Protein–protein interaction network results showed that CASP3, SRC, ANXA5, MMP9, HSP90AA1, IGF1, RHOA, ESR1, EGFR, and CDC42 were identified as key therapeutic targets. GO analysis revealed that these key targets may be involved in the negative regulation of apoptosis and protein phosphorylation. KEGG indicated that pathways in cancer, MAPK signaling pathway, Ras signaling pathway, PI3K-Akt signaling pathway, and HIF-1 signaling pathway were key pathways for renal fibrosis treatment. Molecular docking results showed that CASP3, ANXA5, MMP9, and HSP90AA1 stably bind to diosmin. Diosmin treatment inhibited the protein and mRNA levels of CASP3, MMP9, ANXA5, and HSP90AA1. Network pharmacology analysis and experimental results suggest that diosmin ameliorates renal fibrosis by decreasing the expression of CASP3, ANXA5, MMP9, and HSP90AA1.

**Conclusions:**

Diosmin has a potential multi-component, multi-target, and multi-pathway molecular mechanism of action in the treatment of renal fibrosis. CASP3, MMP9, ANXA5, and HSP90AA1 might be the most important direct targets of diosmin.

## Introduction

The prevalence of chronic kidney disease (CKD) is estimated to be 8%–16% worldwide [1], threatening human health and imposing a heavy economic burden on patients. Renal fibrosis is a pathological feature common to almost all CKD cases that progress to end-stage renal disease [2]. Renal fibrosis is a progressive pathophysiological change from healthy to injured kidney tissues, which are damaged until functional loss. In this process, the kidney is affected by various pathogenic factors such as infection, inflammation, blood circulation disorders, and immune response, resulting in damage and sclerosis of intrinsic cells [3, 4]. In later stages of development, an intense collagen deposition and accumulation occurs, resulting in gradual sclerosis of the renal parenchyma and scarring until the kidney completely loses its organ function [5]. Therefore, early prevention and treatment of renal fibrosis is of great significance to delay the progression of CKD. However, owing to the lack of treatments for renal fibrosis, the search for new therapeutic drugs is an important strategy to prevent renal fibrosis.

Diosmin is a glycosylated polyphenolic flavonoid with antioxidant, anti-inflammatory, and anti-apoptotic pharmacological activities [6]. In recent years, the anti-fibrotic effect of Diosmin has also received increasing attention. Diosmin has been shown to reduce paraquat-induced lung inflammation and fibrosis by increasing glutathione levels and catalase activity and by decreasing hydroxyproline and malondialdehyde levels. In addition, Gerges et al. [7] demonstrated that diosmin ameliorates inflammation, insulin resistance, and liver fibrosis in a rat model of nonalcoholic steatohepatitis. Recently, Geshnigani et al. [8] showed that diosmin ameliorates gentamicin-induced renal injury through antioxidant and anti-inflammatory activities in rats. However, whether diosmin protects against chronic kidney injury by inhibiting interstitial fibrosis remains unclear. The novelty of this study is the first to explore the effect of diosmin on renal fibrosis. This study combined network pharmacology and molecular docking techniques to comprehensively reveal the mechanisms underlying the therapeutic effects of diosmin on renal fibrosis and to predict the key targets and signaling pathways involved.

## Materials and methods

### Query of the targets of diosmin

‘Diosmin’ was used as a search term in PubChem database (https://pubchem.ncbi.nlm.nih.gov/) [9]. The SDF format file of diosmin was obtained and imported into PharmMapper (https://www.lilab-ecust.cn/pharmmapper/) for prediction. This method is based on reverse pharmacophore matching of structural features. The molecular structures of the diosmin components were submitted to the PharmMapper database, and multiple conformations were generated by optimizing the compound structure. The standardized diosmin components of the protein targets were matched with all the human drug targets in the PharmMapper database in UniProt (https://www.uniprot.org/), and protein targets with a parameter matching score (fit) greater than 2 were selected as the component targets.

### Screening the therapeutic targets for renal fibrosis

Human genes associated with renal fibrosis were gathered from three databases: OMIM (https://omim.org/) [10], GeneCards (https://www.genecards.org/) [11], and DisGenet (https://www.disgenet.org/) [12]. The search term ‘renal fibrosis’ was used to retrieve valuable targets from three databases. Finally, the intersection targets of diosmin and renal fibrosis were displayed using a Draw Venn Diagram (http://bioinformatics.psb.ugent.be/webtools/Venn/) [13].

### Protein–protein interaction (PPI)

PPIs of the therapeutic targets of diosmin in the treatment of renal fibrosis were gathered using STRING (https://string-db.org/) [14], a database of known and predicted PPIs that uses bioinformatic strategies to collect information. In this study, we limited the species to ‘Homo sapiens’, collected the PPIs with confidence scores > 0.4 and hid the disconnected nodes in the network.

### Gene ontology (GO) and pathway enrichment analysis

To further explore the mechanisms of diosmin for renal fibrosis treatment, the intersection of targets of diosmin and the common proteins between renal fibrosis were additionally searched using GO (http://geneontology.org/) [15], enrichment and Kyoto Encyclopedia of Genes and Genomes (KEGG) (www.kegg.jp/kegg/kegg1.html) [16–18], and pathway analysis via the online platform DAVID 6.8 (DAVID, https://david.ncifcrf.gov/) [14]. The results obtained by mapping the diosmin targets overlapping renal fibrosis targets were imported into the online software DAVID with H. sapiens set and *P* < 0.05 selected, and the results were analyzed by selecting GO Biological Processes (BP), GO Molecular Functions (MF), GO Cellular Components (CC), and KEGG Pathway for GO and KEGG analysis of the obtained results.

### Molecular docking

The 2D structures of diosmin were downloaded from PubChem (https://pubchem.ncbi.nlm.nih.gov/) [9] and Traditional Chinese Medicine Systems Pharmacology (TCMSP) database; they were then imported to Chemofce 2014 software for the SDF format switching to mol2 format (3D structure). The 3D structures of diosmin were obtained from the RCSB PDB database (http://www.rcsb.org/pdb/) [19] and were input to the PyMol software to separate the original ligand and remove the hydrone, phosphate radical, and other inactive ligands from the proteins [20, 21]. The 3D structures of small molecules (ligands) and target proteins were imported into Auto Dock Vina software to acquire the PDBQT format, and finally docking the molecules with the target proteins; the affinity and the hydrogen bond interaction was shown using PyMol [22].

### Reagent

Diosmin (cat. no.: HY-N0178) was purchased from MedChemExpress (Monmouth Junction, NJ, USA). Human proximal tubular epithelial (HK-2) cells were obtained from American Type Culture Collection (Manassas, VA, USA). Fetal bovine serum was obtained from Millipore Sigma (Burlington, MA, USA). Transforming growth factor-β1 (TGF-β1) was purchased from Proteintech (Chicago, IL, USA). Reverse transcription kit and SYBR Green PCR Master Mix were purchased from Vazyme (Nanjing, China).

### Cell culture and treatment

To assess cell viability, HK-2 cells were cultured at 1 × 10^4^ cells/well in 96-well plates. After treatment with diosmin for 3 h, they were incubated with 0.5 mg/mL of 3-(4,5-Dimethylthiazol-2-yl)-2,5-diphenyltetrazolium bromide solution (100 μL/well) for 4 h. After washing with 1 × PBS, dimethyl sulfoxide (150 μL/well) was added to dissolve purple crystals. The absorbance of the samples was measured at 570 nm using a microplate reader (BioTek Instruments, VT, USA). Furthermore, diosmin was administered to TGF-β1 (5 ng/mL)-stimulated HK-2 cells. The effects of diosmin on core genes were observed in HK-2 cells.

### Real-time PCR

Total RNA was extracted from HK-2 cells or mouse kidney tissues using TRIzol® reagent. Complementary DNA was synthesized using Hiscript Q RT SuperMix and used with the qPCR (+ gDNA wiper) reverse transcriptase kit (Vazyme). The sequences of the used primes for Real-time PCR are listed in Table 1. Messenger (m)RNA expression in the corresponding samples was normalized to GAPDH mRNA.

**Table 1:** Primer sequences used for RT-qPCR

Gene	Forward (5’ to 3’)	Reverse (5’ to 3’)
HSP90AA1	CCAGTTCGGTGTTGGTTTTTAT	CAGTTTGGTCTTCTTTCAGGTG
Annexin 5	GTTCCATGGGCGCACAGGTTCTCAGAGGCA	TCCGCTCGAGTTAGCAGTCATCTTCTCCACAGAGCA
CASP3	CCAAAGATCATACATGGAAGCG	CTGAATGTTTCCCTGAGGTTTG
MMP9	CAGTACCGAGAGAAAGCCTATT	CAGGATGTCATAGGTCACGTAG
GAPDH	TGATGACATCAAGAAGGTGGTGAAG	TCCTTGGAGGCCATGTGGGCCAT

### Statistical analyses

Values are presented as the mean ± SEM. Quantitative data were tested for normality. Two-tailed unpaired t-test and one-way ANOVA were used to compare differences between two groups and multiple groups, respectively. Prism 9.0 (GraphPad, San Diego, CA, USA) was used for the statistical analyses. *P* < 0.05 was designated significant.

## Results

### The targets of diosmin and therapeutic targets for renal fibrosis

**Fig. 1:**
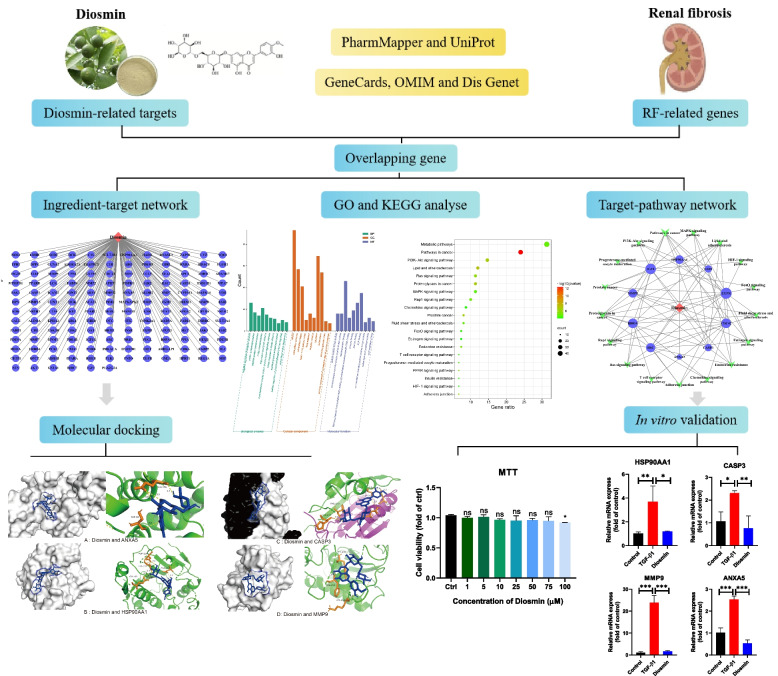
Flowchart of network pharmacology analysis of Diosmin against renal fibrosis

**Fig. 2:**
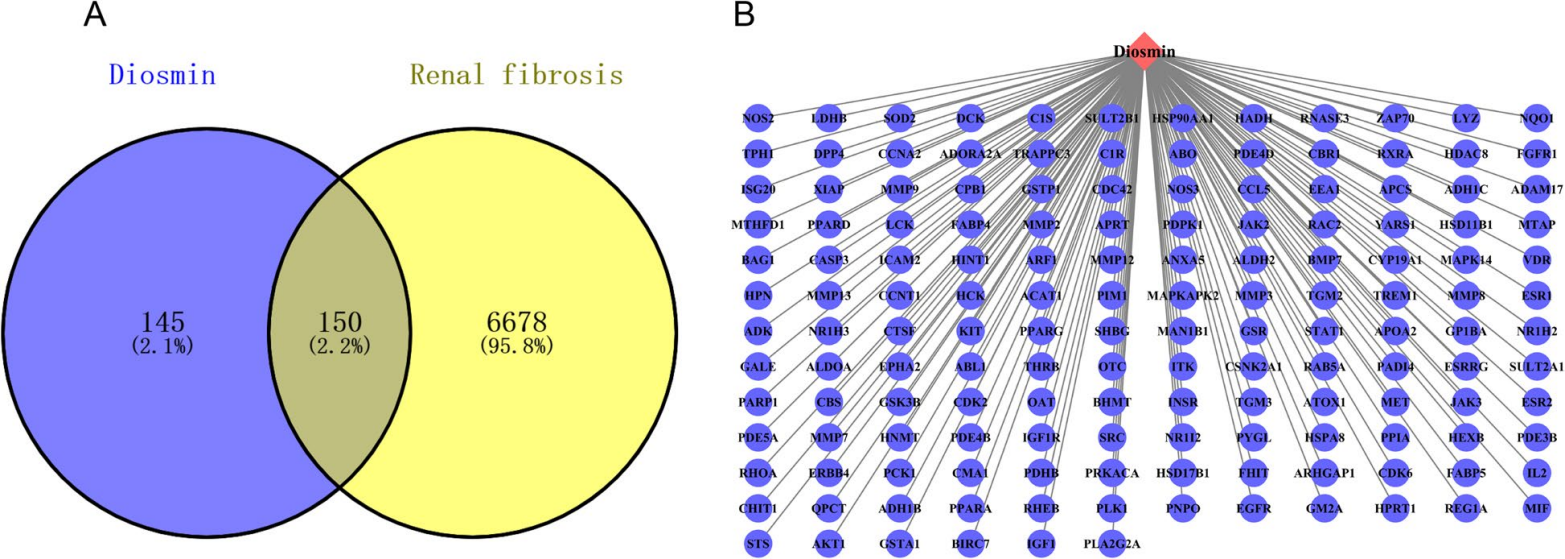
The 150 overlapping genes between diosmin and renal fibrosis targets. **a** Venn diagram of Diosmin-related targets and renal fibrosis-related targets. **b** The ingredient-target network of Diosmin against renal fibrosis

Figure 1 shows the flow of the network pharmacological analysis of Diosmin against renal fibrosis. The chemical formula of the diosmin molecule C_28_H_32_O_15_ was obtained from PubChem, and the targets related to diosmin were screened using the PharmMapper database and corrected using the UniProKB tool in the UniProt database. A total of 295 potential targets, including alpha1antitrypsin (A1AT), caspase 3 (CASP3), mitogen-activated protein kinase 14 (MAPK14), and matrix metalloprotein (MMP9), were screened after removing targets that were unrelated to humans and those without correspondence or duplication. A total of 266, 570, and 6546 target genes closely related to renal fibrosis were screened in the three commonly used databases of GeneCards, OMIM, and Dis Genet, respectively. A total of 6828 genes were identified after duplicates were eliminated. These results suggest that numerous factors cause renal fibrosis, and the pathogenesis is complex. Gene datasets obtained from the screening of renal fibrosis-related targets and diosmin component-related targets were imported into an online Venn diagram, and a total of 150 intersecting targets were obtained (Fig. 2).

### Drug-disease target PPI network

**Fig. 3:**
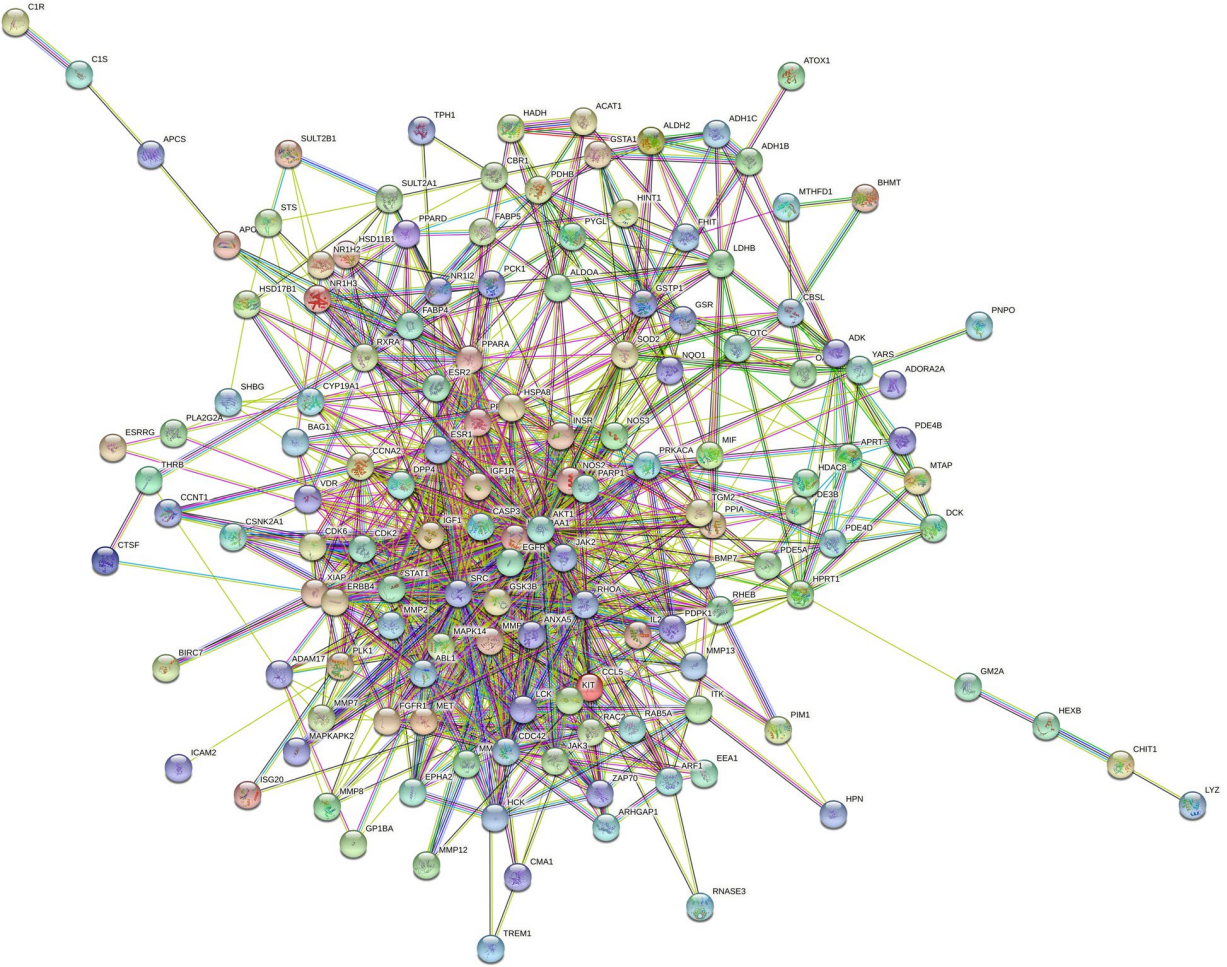
PPI network of intersection targets of 150 overlapping genes

**Fig. 4:**
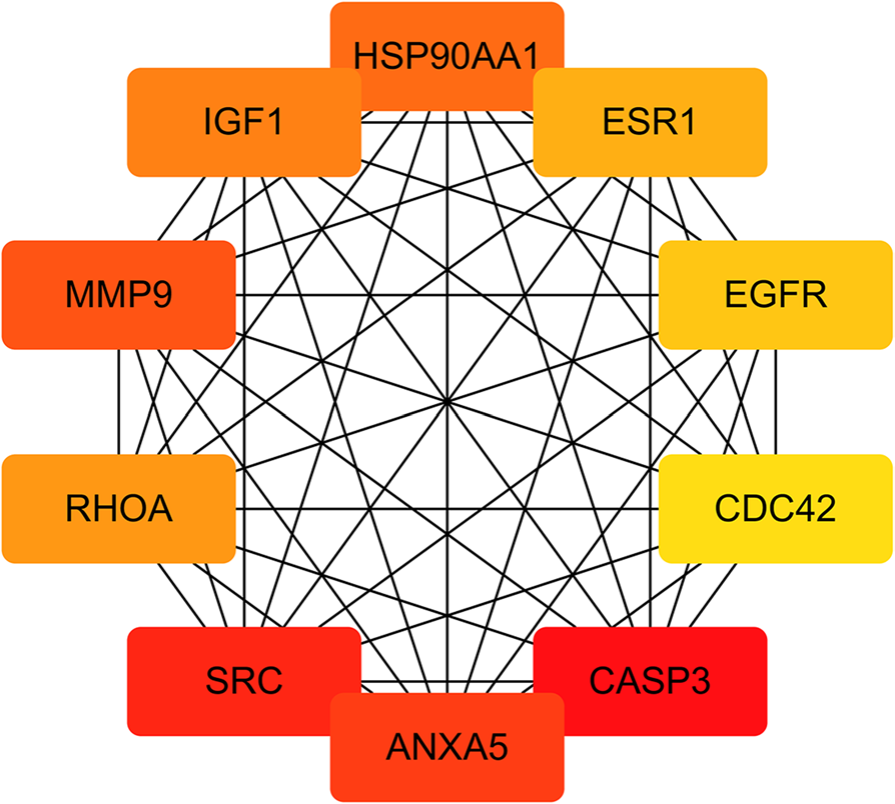
Ten high-freedom overlapping genes

A total of 150 hub genes were screened by mapping the diosmin component targets to each other and to the renal fibrosis disease targets. The 150 targets were imported into the STRING database and imported into Cytoscape for visualization and analysis, and a network consisting of 112 nodes and 721 edges was obtained. The network density was 0.588, and the average node degree was 12.9. When the network density is greater than 0.5, and the average node degree is greater than 3, the network has a good correlation (Fig. 3). In the network, the top ten targets with high degrees of freedom were CASP3, SRC proto-oncogene, non-receptor tyrosine kinase (SRC), annexin A5 (ANXA5), matrix metallopeptidase 9 (MMP9), heat shock protein 90 alpha family class A member 1 (HSP90AA1), insulin-like growth factor 1 (IGF1), ras homolog family member A (RHOA), estrogen receptor 1 (ESR1), epidermal growth factor receptor (EGFR), and cell division cycle 42 (CDC42) (Fig. 4). The related gene targets focused on apoptosis and inflammatory pathways.

### GO and KEGG pathway enrichment

**Fig. 5:**
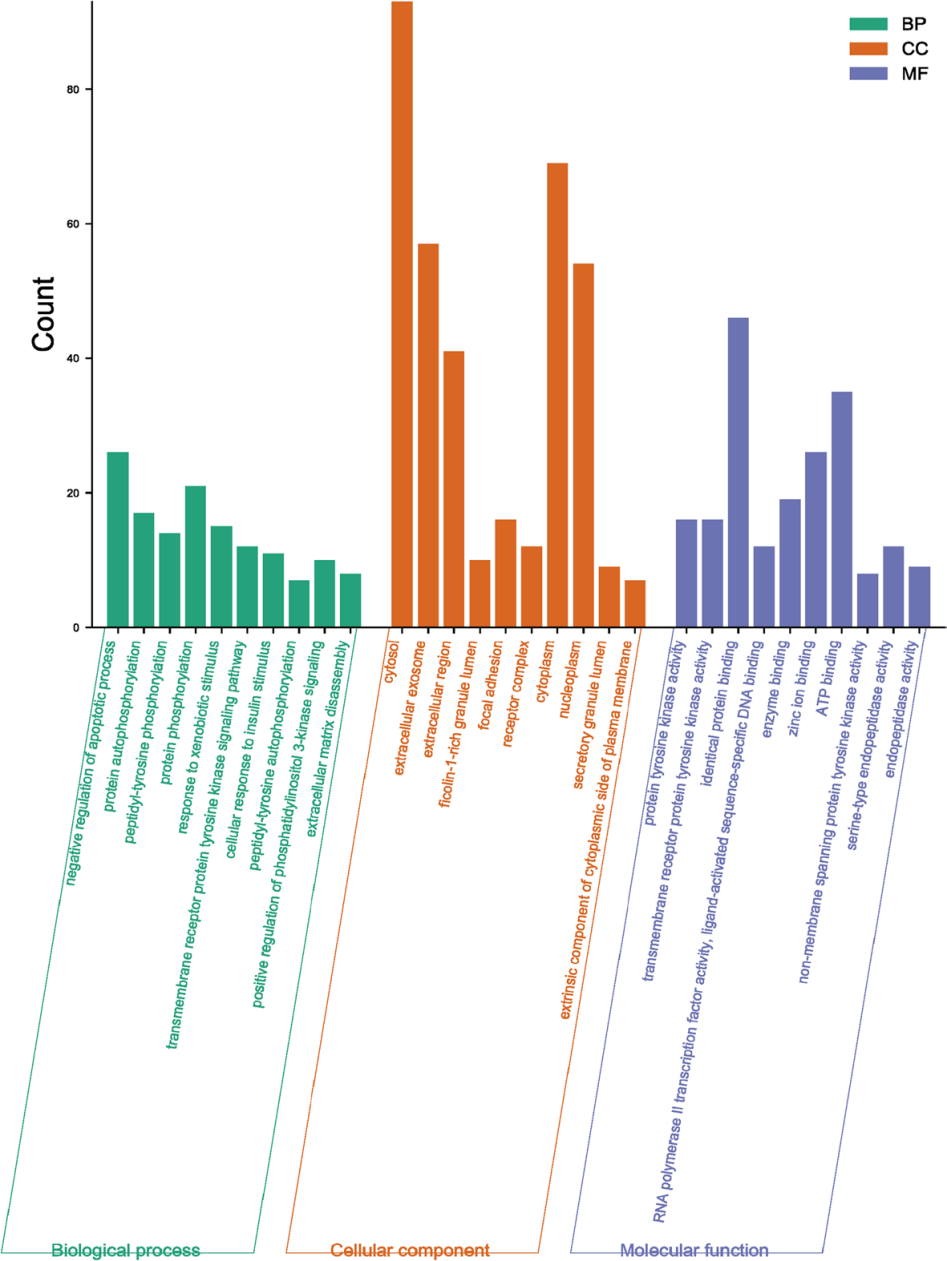
GO (BP, MF, CC) analyses of therapeutic target genes of diosmin for treatment of renal fibrosis. Each bar represents a GO term on the horizontal axis. The number of genes enriched in each term is shown on the vertical axis

**Fig. 6:**
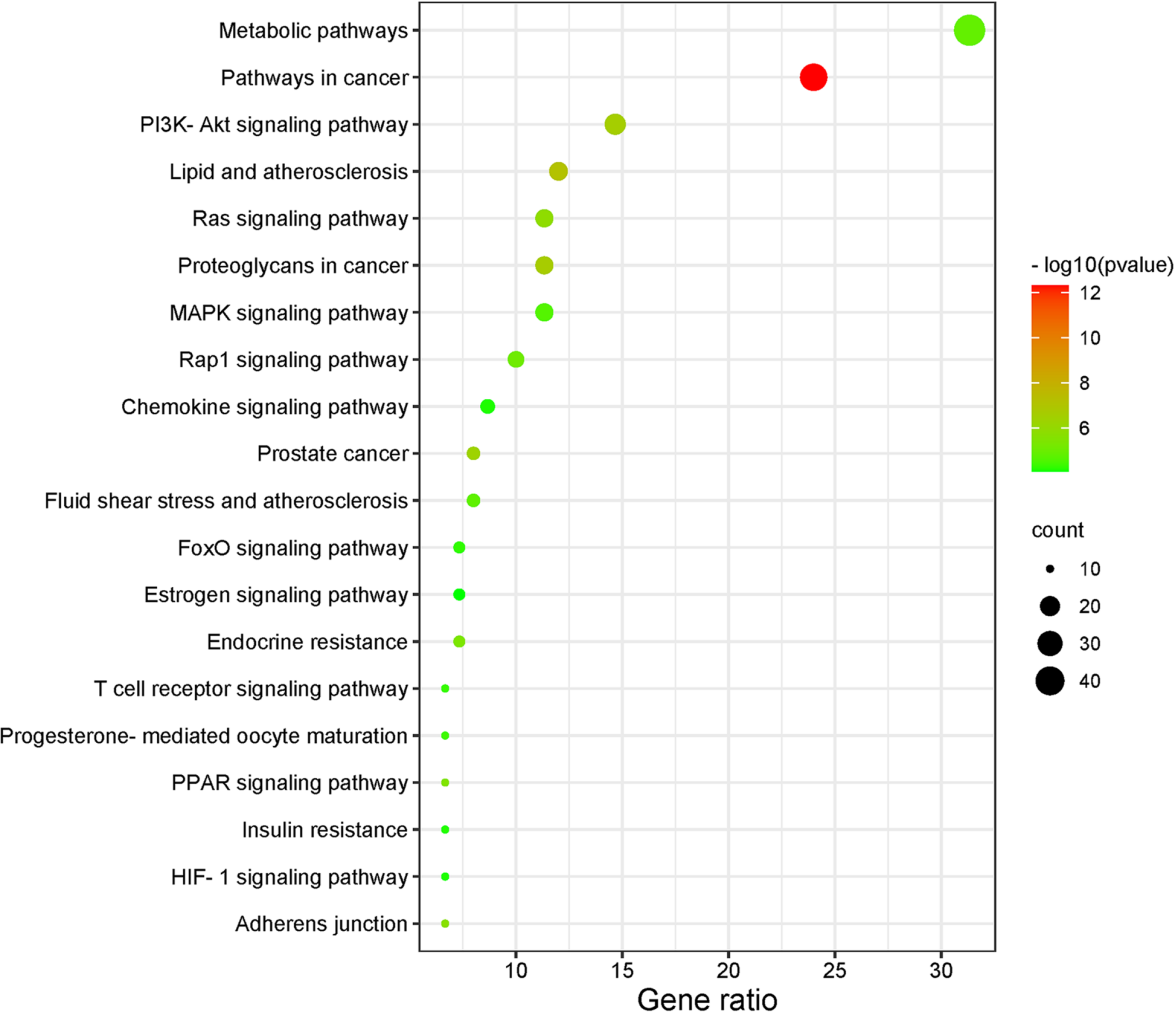
KEGG analyses of the therapeutic target genes of diosmin for treatment of renal fibrosis. Each bubble represents a KEGG pathway on the vertical axis. The gene ratio is shown on the horizontal axis. The size of each bubble indicates the number of genes enriched in each KEGG pathway. Larger bubbles indicate more genes involved in the pathway. The color of each bubble represents the adjusted P-value of each KEGG pathway, with redder color indicating smaller adjusted *P*-value

GO and KEGG pathway enrichment results were plotted as bar graphs and bubble plots by gene number for visual analysis. A total of 291 BP, 44 CC, 85 MF, and 98 KEGG signaling pathways were identified [16–18]. GO analysis showed that diosmin mainly plays a role in the BPs of apoptosis negative regulation, protein phosphorylation, protein autophosphorylation, peptidyl-tyrosine phosphorylation, transmembrane receptor protein tyrosine kinase signaling pathway, response to foreign body stimulation, cellular response to insulin stimulation, and positive regulation of phosphatidylinositol 3-kinase signaling. Apoptosis was correlated with the development of renal fibrosis, while protein phosphorylation was associated with the regulation of protein activity. CC was mainly enriched in the cytoplasm and nucleoplasm. MF analysis showed an association between homologous protein binding and ATP binding (Fig. 5). KEGG enriched 98 signaling pathways (*P* < 0.05), including cancer, lipids and atherosclerosis, proteoglycans in cancer, fluid shear stress and atherosclerosis, endocrine resistance, and Ras-related protein 1 (Rap1), rat sarcoma (Ras), Phosphoinositide-3 kinase (PI3K)/Akt, chemokine, Mitogen-activated protein kinase (MAPK), Forkhead Box Protein O (FoxO), T-cell receptor, hypoxia-inducible factor-1 (HIF-1), and estrogen signaling pathways (Fig. 6). Among these, the MAPK, Ras, PI3K/Akt, FoxO, and HIF-1 signaling pathways were closely related to renal fibrosis.

### Molecular docking

**Fig. 7:**
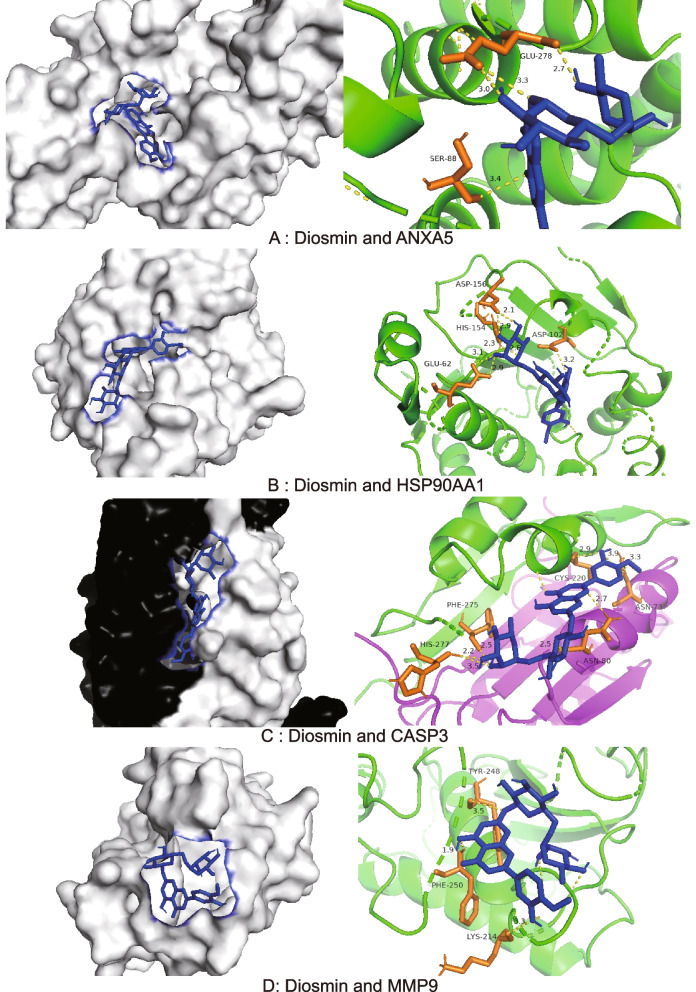
Molecular docking pattern and mapping surface showing molecules occupying the active pocket of proteins

Diosmin and the top ten targets ranked by degree were selected for molecular docking (Table 2). The binding capacity score is an important indicator of the ability of the receptor and ligand to bind to each other; the lower the binding capacity score, the more stable the complex formed [23–25]. Following the convention, a binding capacity between the tested molecules and proteins was assumed to exist when the binding energy score was greater than 4.25. Scores greater than 5.0 indicate relatively high binding affinity [26]. Hence, the selected diosmin was docked with MMP9, ANXA5, CASP3, and HSP90AA1 using AutoDock according to its binding capacity (Fig. 7).

**Table 2:** Information on the molecular docking of diosmin with the top 10 targets

No	Protein	Ligand	Binding capacity (Kcal / mol)	Protein	Ligand	Binding capacity (Kcal / mol)
1	ANXA5	diosmin	-5.17	CASP3	diosmin	-6.28
2	IGF1	diosmin	-4.14	RHOA	diosmin	-4.78
3	EGFR	diosmin	-2.67	MMP9	diosmin	-5.73
4	SRC	diosmin	-3.74	ESR1	diosmin	-2.45
5	HSP90AA1	diosmin	-5.76	CDC42	diosmin	-3.28

### Effect of diosmin on the expression of target genes in HK-2

**Fig. 8:**
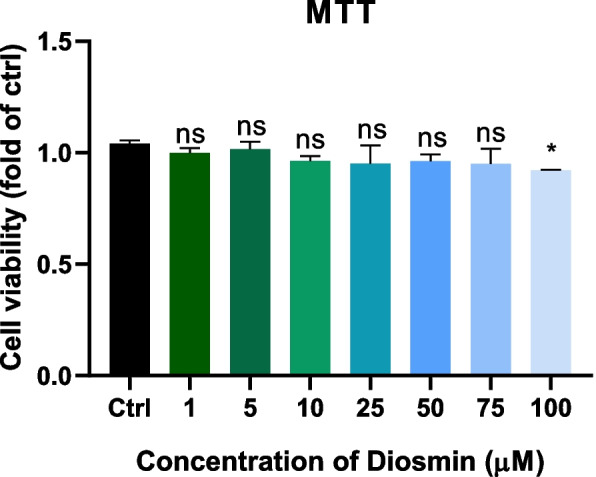
MTT assay detects the toxicity of diosmin on HK-2 cells

**Fig. 9:**
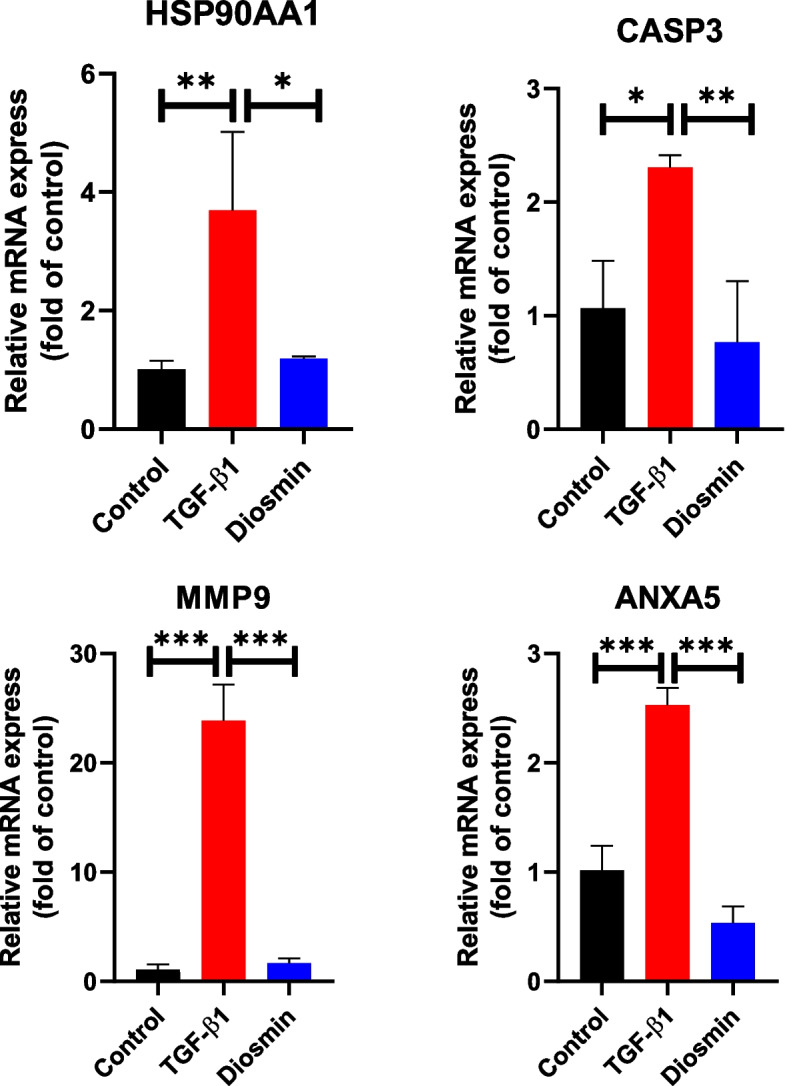
The mRNA expression levels of CASP3, MMP9, ANXA5, and HSP90AA1 in different groups of HK-2 cells

We further investigated the anti-fibrotic effect of diosmin on HK-2 cells. Diosmin treatment (0, 1, 5, 10, 25, 50, or 75 μM) of HK-2 cells did not significantly inhibit the viability (Fig. 8). Cell viability was slightly inhibited at a concentration of 100 μM. Therefore, we treated the cells with 75 μM diosmin. Compared with the TGF-β1 group, the mRNA expression of ANXA5, CASP3, MMP9, and HSP90AA1 was significantly decreased in the diosmin group (Fig. 9).

## Discussion

Renal fibrosis is a common pathological feature in the terminal stages of various CKDs and is closely related to their prognosis [27]. Early prevention and treatment of renal fibrosis are of great significance in delaying the progression of chronic kidney disease. However, there is a lack of effective drugs for the treatment of renal fibrosis. Network pharmacology is an emerging discipline built on the disease and pharmacogenetic level [28]. By predicting new drug targets, identifying modes of action, and exploring new drugs, network pharmacology opens a new research paradigm of complex web-like relationships between multiple targets and multiple diseases [29]. In this study, we used a systematic network pharmacological approach to explore the potential molecular mechanisms of action of diazepine in renal fibrosis. Through PPI network and molecular docking module analyses, CASP3, MMP9, ANXA5, and HSP90AA1 were identified as major pivotal gene targets in renal fibrosis. Additionally, these four core genes were validated in HK-2 cells.

We found that core genes are mainly involved in the biological process of apoptosis. The protein encoded by CASP3 is a cysteine-aspartic acid protease that plays a central role in the execution phase of cell apoptosis. Caspase 3 is also a critical upstream regulator in the development of renal fibrosis [30]. Caspase 3 inhibitors have been shown to reduce renal interstitial fibrosis in diabetic nephropathy or obstructive nephropathy [31, 32]. Annexin 5 is a phospholipase A2 and protein kinase C inhibitory protein with calcium channel activity that has a potential role in cellular signal transduction, inflammation, growth, and differentiation [33]. Annexin V binds specifically to phosphatidylserine (PS) and is often used as a sensitive indicator of early apoptosis of cells. To date, no studies have proved a direct correlation between this protein and renal fibrosis. Matrix metalloproteinases (MMPs) belong to the family of zinc-dependent endoproteases. Their functions are based on remodeling and degradation of protein components of the extracellular matrix (ECM) [34]. Tan et al. suggested that MMP-9 directly contributes to the pathogenesis of renal fibrosis via induction of tubular cell epithelial-mesenchymal transition and osteopontin cleavage, which in turn recruits macrophages [35]. HSP90AA1 (heat shock protein 90 alpha family class A member 1) is a highly conserved molecular chaperone ubiquitously expressed in eukaryotic cells [36]. Research has suggested that myocardial fibroblasts from HSP90AA1 knock-out mice exhibited low collagen production [37]. Therefore, the core genes CASP3, ANXA5, MMP9, and HSP90AA1 may play key roles in the inhibition of the occurrence and development of renal fibrosis.

The core genes SRC, IGF1, RHOA, ESR1, EGFR and CDC42 may play a key role in the inhibition of the development and progression of renal fibrosis by diosgenin. This SRC gene may play a role in the regulation of embryonic development and cell growth. The protein encoded by this gene is a tyrosine-protein kinase whose activity can be inhibited by phosphorylation by c-SRC kinase. SRC kinase plays an important role in cell proliferation, differentiation, migration, and immune response, and it is a critical mediator of renal fibrosis [38, 39]. Activation of FXR has been found to attenuate renal fibrosis by inhibiting the phosphorylation of SRC, regulating the hippo pathway, and modulating the phosphorylation and localization of YAP [40]. Both IGF-1 and IGF-1 receptor levels are increased in glomeruli of diabetic rats, and this growth factor may be profibrotic [41, 42]. RhoA is one of the most studied Rho GTPases and is involved in a variety of cellular activities [43]. Research showed that extracellular vesicles produced by bone marrow mesenchymal stem cells attenuate renal fibrosis, in part by inhibiting the RhoA/ROCK pathway [44]. The ESR1 gene encodes the estrogen receptor. Research showed that Tamoxifen, a selective estrogen receptor modulator exhibits antifibrotic effects in the L-NAME model of hypertensive nephrosclerosis [45]. The protein encoded by the EGFR gene is a transmembrane glycoprotein that is a member of the protein kinase superfamily. In fibrotic disease induced by AKI or CKD, EGFR is frequently in a state of continuous activation in proximal tubule cells, which contributed to the progression of renal fibrosis in renal injury [46]. The protein encoded by the CDC42 gene is a small GTPase of the Rho-subfamily. Research indicated that ARAP1‐AS2/ARAP1 may participate in cytoskeleton rearrangement and EMT processes in HK‐2 cells through increased CDC42-GTP levels and induced renal Fibrosis [47].

According to the KEGG terms, the therapeutic targets of diosmin against renal fibrosis were mainly associated with the MAPK, Ras, PI3K-Akt, FoxO, and HIF-1 signaling pathways. MAPKs are a group of protein kinases containing three subfamilies: c-Jun N-terminal kinase (JNK), extracellular signal-regulated kinase (ERK), and p38 [48]. MAPKs regulate many cellular functions, including proteasomal degradation [49]. ERK, p38, and JNK MAPK pathways are involved in kidney injury and fibrosis [26, 50]. Ras monomeric GTPases play a significant role in controlling proliferation, differentiation, and cell death. Research has shown that the CF ethanol extract may ameliorate renal fibrosis via the Wnt/β-catenin/RAS pathway [51]. An increasing number of reports have shown that the PI3K/AKT signaling pathway may play a crucial role in renal fibrosis and dysfunction by regulating various proteins [52]. Research has suggested that Chlorogenic Acid exerts protective effects against renal fibrosis by inhibiting PI3K/Akt signaling [53]. Forkhead homeobox type O (FoxO) transcription factors mediate cellular responses to oxidative stress and have been implicated in many ROS-regulated processes [54]. The PI3K/Akt/FoxO signaling pathway may play a role in ROS-mediated diseases, as shown by research in which Tempol attenuated renal fibrosis in mice with unilateral ureteral obstruction [55]. Hypoxia-inducible factors (HIFs), critical nuclear transcription factors, are involved in maintaining O_2_ homeostasis [56]. Based on the difference in the α-subunits, HIFs are divided into three subtypes: HIF-1, HIF-2, and HIF-3. Reportedly, oxidative factors induced renal fibrosis by regulating the expression and activity of HIF-1 via PHD, ERK, and PI-3 K/AKT pathways [57, 58]. Therefore, the MAPK, Ras, PI3K-Akt, FoxO, and HIF-1 signaling pathways are closely related to the occurrence and development of renal fibrosis.

We explored the potential molecular mechanism of action of diosmin in the treatment of renal fibrosis from a comprehensive and systematic perspective, and our results provide a theoretical basis for further experimental studies. Network pharmacology analysis and molecular docking technology were used to explore the potential mechanism of action of diosmin against renal fibrosis, and the key therapeutic targets of diosmin were identified as CASP3, MMP9, ANXA5, and HSP90AA1. The mechanism of action of diosmin in the treatment of renal fibrosis may be related to the regulation of biological pathways such as cell apoptosis and inflammation. This study provides a theoretical basis for the treatment of diosmin-induced renal fibrosis. A limitation of this study is the lack of additional experimental areas to validate our findings. However, the specific mechanisms involved in this process require further exploration. Future studies should conduct well-designed in vivo and in vitro experiments to further explore the specific mechanisms.

## Conclusion

Diosmin has a potential multi-component, multi-target, and multi-pathway molecular mechanism of action in the treatment of renal fibrosis. CASP3, MMP9, ANXA5, and HSP90AA1 might be the most important direct targets of diosmin. The mechanism of action may be related to the MAPK, Ras, PI3K-Akt, FoxO, and HIF-1 signaling pathways. This study provides a basis for further studies on diosmin in the treatment of renal fibrosis.

## Data Availability

The datasets analyzed during the current study are available in the following public available databases. PubChem database (https://pubchem.ncbi.nlm.nih.gov/), UniProt (https://www.uniprot.org/), PharmMapper (https://www.lilab-ecust.cn/pharmmapper/), GeneCards (https://www.genecards.org/), OMIM (https://omim.org/), DisGenet (https://www.disgenet.org/), STRING (https://string-db.org/), GO (http://geneontology.org/), KEGG (www.kegg.jp/kegg/kegg1.html), DAVID 6.8 (https://david.ncifcrf.gov/) and RCSB PDB database (http://www.rcsb.org/pdb/).
